# Impact of diabetes and hypertension on cardiovascular outcomes in patients with coronary artery disease receiving percutaneous coronary intervention

**DOI:** 10.1186/s12872-016-0454-5

**Published:** 2017-01-05

**Authors:** Mao-Jen Lin, Chun-Yu Chen, Hau-De Lin, Han-Ping Wu

**Affiliations:** 1 Division of Cardiology, Department of Medicine, Taichung Tzu Chi Hospital, Buddhist Tzu Chi Medical foundation, Taichung, Taiwan; 2 Department of Medicine, School of Medicine, Tzu Chi University, Hualien, Taiwan; 3 Division of Emergency Medicine, Department of Pediatrics, Changhua Christian Hospital, Changhua, Taiwan; 4 School of Medicine, Kaohsiung Medical University, Kaohsiung, Taiwan; 5 Division of Pediatric General Medicine, Department of Pediatrics, Chang Gung Memorial Hospital at Linko, No. 5, Fu-Hsin Street, Kweishan, Taoyuan, 33057 Taiwan; 6 College of Medicine, Chang Gung University, Taoyuan, Taiwan

**Keywords:** PCI, Coronary artery disease, Diabetes, Hypertension

## Abstract

**Background:**

Percutaneous coronary intervention (PCI) is a necessary procedure commonly performed for patients with coronary artery disease (CAD). However, the impact of diabetes and hypertension on long-term outcomes of patients after receiving PCI has not yet been determined.

**Methods:**

The data of 1234 patients who received PCI were collected prospectively, and patients were divided into four groups, including patients with and without DM and those with either DM or hypertension alone. Baseline characteristics, risk factors, medications and angiographic findings were compared and determinants of cardiovascular outcomes were analyzed in patients who received PCI.

**Results:**

Patients with DM alone had the highest all-cause mortality (*P* < 0.001), cardiovascular mortality and myocardial infarctions (MI) (both *P* < 0.01) compared to the other groups. However, no differences were found between groups in repeat PCI (*P* = 0.32). Cox proportional hazard model revealed that age, chronic kidney disease (CKD), previous MI and stroke history were risk factors for all-cause mortality (OR: 1.05,1.89, 2.87, and 4.12, respectively), and use of beta-blockers (BB) and statins reduced all-cause mortality (OR: 0.47 and 0.35, respectively). Previous MI and stroke history, P2Y12 inhibitor use, and syntax scores all predicted CV mortality (OR: 4.02, 1.89, 2.87, and 1.04, respectively). Use of angiotensin converting enzyme inhibitors (ACEI), beta-blockers (BB), and statins appeared to reduce risk of CV death (OR: 0.37, 0.33, and 0.32, respectively). Previous MI and syntax scores predicted MI (OR: 3.17 and 1.03, respectively), and statin use reduced risk of MI (OR: 0.43). Smoking and BB use were associated with repeat PCI (OR: 1.48 and 1.56, respectively).

**Conclusions:**

After PCI, patients with DM alone have higher mortality compared to patients without DM and hypertension, with both DM and hypertension, and with hypertension alone. Comorbid hypertension does not appear to increase risk in DM patients, whereas comorbid DM appears to increase risk in hypertensive patients.

**Trial registration:**

REC103-15 IRB of Taichung Tzu-chi Hospital

## Background

Percutaneous coronary intervention (PCI) refers to coronary revascularization through a trans-arterial approach using a various spectrum of devices. PCI is necessary and commonly performed for patients with coronary artery disease (CAD). Clinical outcomes of patients with PCI may include myocardial infarction (MI), revascularization and mortality [1]. Major risk factors such as diabetes mellitus (DM), hypertension, dyslipidemia and smoking can also affect outcomes in CAD patients receiving PCI.

The impact of DM and hypertension on outcomes in patients with acute coronary syndrome (ACS) receiving PCI has been well studied. Hypertension did not affect short-and long term mortality in patients with ST elevation myocardial infarction (STEMI) receiving PCI [2, 3]. However, hypertension was the only independent long-term predictor of mortality in patients with unstable angina (US) receiving coronary stenting [4]. Insulin-treated diabetes mellitus (ITDM) was a strong predictor for long-term mortality when compared with non-DM or non-ITDM patients [5]. After receiving PCI, diabetic patients with ACS had worse short- and mid-term outcomes than non-diabetes patients with ACS [6–9]. For ACS patients with both DM and hypertension, the combination of DM and hypertension appeared to be strongly associated with mortality than in patients with DM or hypertension alone [10]. For patients with stable CAD after receiving PCI, diabetes was still an adverse predictor for mid-term outcomes [11, 12].

However, the combined effect of diabetes and hypertension on long-term outcomes in patients receiving PCI remains obscure. For this reason, the aim of the present study was to clarify and compare the long-term outcomes in four groups of patients: those with diabetes and hypertension, those with only DM, those with only hypertension, and those without either DM or hypertension. We also further analyzed the predictors for adverse clinical outcomes among these four groups.

## Methods

### Study population

This prospective cohort study was conducted via medical record survey from 2007 through 2014. We recruited consecutive PCI patients aged 20 to 90 years from the inpatient clinic at Taichung Tzu Chi Hospital, Taiwan. The patients were divided into four groups: patients without DM and hypertension, patients with DM alone, patients with hypertension alone, and patients with both DM and hypertension. Patients with scheduled PCI and malignancy were excluded. Most patients were followed regularly via the outpatient department (OPD). For the few patients lost to follow-up at the OPD, a telephone call was usually used to contact the patients themselves or their families. For each patient, a survey on cardiovascular mortality (CV mortality), all-cause mortality, MI and repeated PCI procedures was completed at the end of the study. The Institutional Review Board and ethics committee approved the study protocol and signed informed consent was obtained from all study participants.

### Data collection, measurements and analysis

Data of body habitus, baseline biochemical data, hemodynamic data on cardiac catheterization, exposed risk factors and differences between treatment strategies such as drug medications or invasive procedures (balloon angioplasty, bare metal stent deployment or drug-eluting stent deployment) were all collected for analysis. The measurements of body parameters included body height, body weight, and body mass index (BMI). The following baseline biochemical data were collected: fasting plasma glucose, creatinine, total cholesterol, high density lipoprotein-cholesterol (HDL-C), low density lipoprotein-cholesterol (LDL-C) and serum triglyceride level. For hemodynamic data, we collected central aortic pressure (CAP) and left ventricular ejection fraction (LVEF). CAP was measured via pigtail catheter while performing coronary angiography. Angiographic findings, including number of diseased vessels and lesion locations were recorded, and lesion severity and complexity were evaluated by Synergy between Percutaneous Coronary Intervention with Taxus and Cardiac Surgery score (Syntax Score) [13]. The left ventricular ejection fraction was estimated via angiographic ventriculography or scintigraphic ventriculography. Diabetes was defined as a fasting plasma glucose level of more than 126 mg/dL, a causal plasma glucose level greater than 200 mg/dl or hemoglobin A1c (HbA1c) level of more than 6.5% [14]. Hypercholesterolemia was defined as a serum cholesterol level of more than 200 mg/dL or an LDL-C level of more than 100 mg/dL. Chronic kidney disease (CKD) was defined as an estimated glomerular filtration rate (eGFR) of less than 60 ml/min/1.73 m^2^, which is equal to or more than stage III chronic kidney disease (CKD) [15]. Previous MI history was defined as a history of MI prior to index PCI, accompanied by a threefold elevation of cardiac enzymes from the baseline value. Related clinical parameters, including baseline characteristics, hemodynamic data, major risk factors, angiographic findings and invasive strategies, were compared between the four groups. Clinical outcomes, including cardiovascular mortality, all-cause mortality, de novo MI, and repeated PCI were also analyzed in the four groups. Risk factors for adverse clinical outcomes were analyzed to compare differences between the four groups.

### Statistical analysis

Statistical analysis was used primarily to compare differences between the four groups. Analysis of variance (ANOVA) was used to evaluate continuous variables, whereas chi-squared test or Fisher’s exact test were used to evaluate categorical variables. The log-rank test and Kaplan-Meier curves were used for survival analysis. The Cox proportional hazards model was used to eveluate effects of the independent variables on hazards. *P* values of less than 0.05 were considered statistically significant. All analyses were performed using the statistical package SPSS for Windows (Version 22.0 SPSS Inc., Chicago, IL, USA).

## Results

During the study period, a total of 1234 patients who received the PCI procedure were enrolled. Of these, 359 patients in the control group had neither DM nor hypertension, 178 patients had DM alone, 382 patients had hypertension alone, 315 patients had both DM and hypertension. No differences were found in mean follow-up time between the four groups (control group: 173.8 ± 106.8 weeks, DM alone: 155.4 ± 104.8 weeks, Hypertension alone: 168.8 ± 99.7 weeks, both DM and hypertension: 160.9 ± 99.0 weeks, *P* = 0.170).

Patients’ baseline clinical characteristics are listed in Table 1. No significant age differences were found among the four groups (*P* = 0.11). For body habitus parameters, patients with hypertension alone and patients with both DM and hypertension had higher BMI values compared with the other two groups (*P* < 0.01). For hemodynamic parameters, patients with both DM and hypertension had the highest central systolic pressure (CSP) compared with the other groups (*P* < 0.01), whereas patients with hypertension alone had the highest central diastolic pressure (CDP) compared with the other groups (*P* < 0.01). For baseline biochemistries, patients with DM alone had the lowest cholesterol and HDL-C levels (*P* = 0.03 and *P* < 0.01, respectively), while patients with both DM and hypertension had the poorest renal function (*P* < 0.01).

**Table 1:** General characteristics of the study population

	Study Groups				*P* value
	Control (*N* = 359)	DM alone (*N* = 178)	HT alone (*N* = 382)	DM and HT (*N* = 315)	
Age (years)	62.1 ± 12.7	62.3 ± 10.8	63.9 ± 11.5	64.4 ± 10.7	0.10
Weight (kg)	66.3 ± 11.7	67.5 ± 13.2	68.5 ± 13.0	68.6 ± 13.8	0.08
Height (cm)	163.1 ± 7.9	161.8 ± 8.7	162.1 ± 8.6	161.9 ± 8.8	0.18
BMI (kg/m^2^)	24.8 ± 3.5	25.7 ± 4.1	26.0 ± 4.0	26.0 ± 4.0	<0.01
CSP	126.9 ± 20.8	130.0 ± 20.0	141.9 ± 21.6	145.7 ± 25.6	<0.01
CDP	71.8 ± 12.2	70.5 ± 12.4	76.1 ± 13.0	72.8 ± 13.6	<0.01
Cholesterol (mg/dl)	182.7 ± 44.2	171.3 ± 42.2	182.1 ± 42.0	179.1 ± 49.0	0.03
HDL (mg/dl)	41.5 ± 15.8	36.8 ± 14.4	40.2 ± 16.4	37.1 ± 15.4	<0.01
TG (mg/dl)	149.9 ± 110.9	153.8 ± 102.0	155.1 ± 95.1	177.3 ± 121.6	<0.01
LDL (mg/dl)	111.3 ± 39.7	103.8 ± 34.7	110.9 ± 38.2	106.2 ± 39.7	0.07
Serum creatinine (mg/dl)	1.3 ± 1.3	1.8 ± 2.2	1.6 ± 1.9	2.3 ± 2.9	<0.01
Uric acid (mg/dl)	6.6 ± 2.0	6.6 ± 2.2	6.5 ± 2.1	7.2 ± 2.0	0.06

*DM alone* diabetes alone, *HT alone* hypertension alone, *DM and HT* both DM and hypertension, *BMI* body mass index, *Central SP* central aortic systolic pressure, *Central DP* central aortic diastolic pressure, *HDL* high-density lipoprotein cholesetrol, *LDL* low- density lipoprotein cholesterol, *TG* triglyceride

**Fig. 1:**
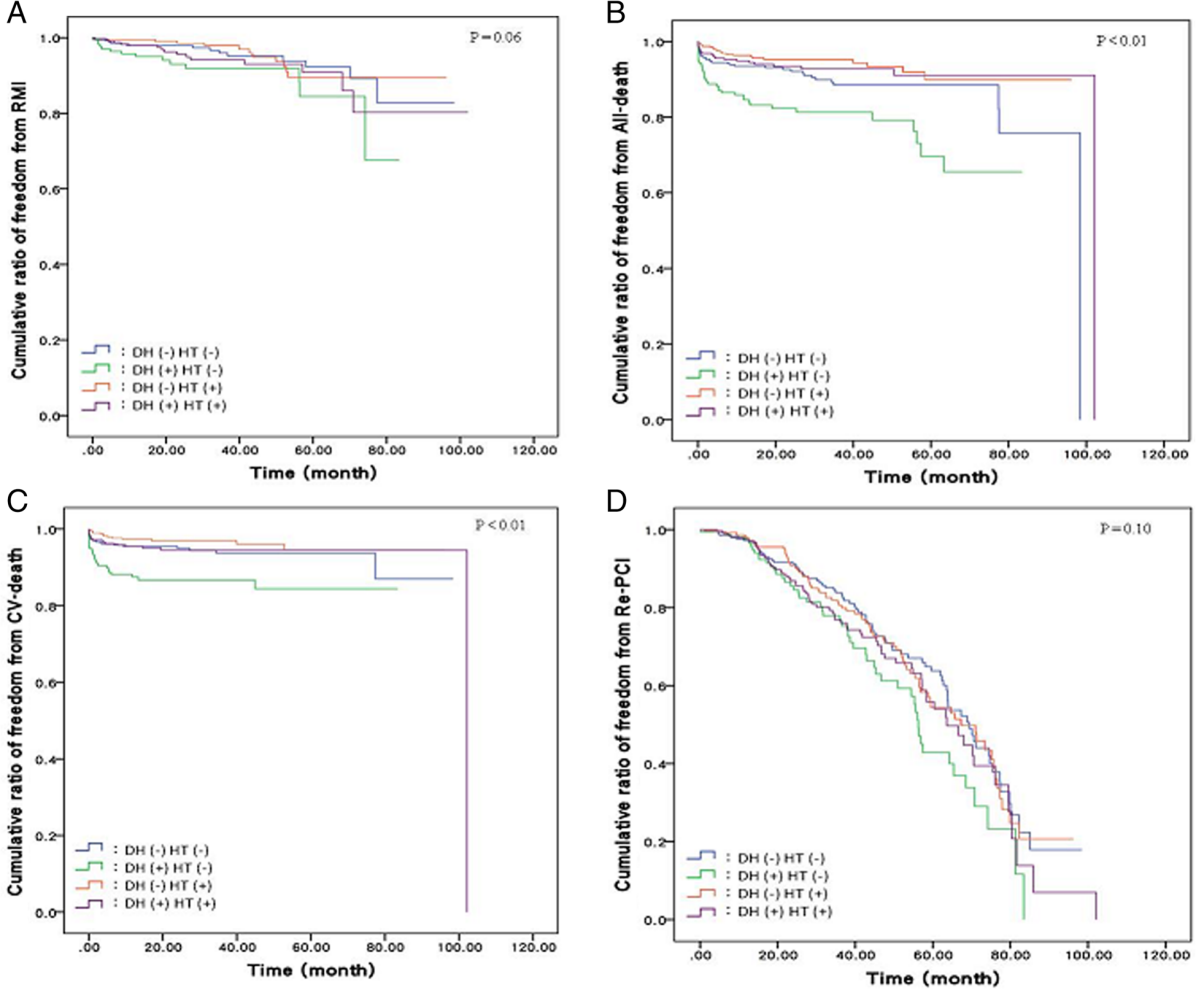
**a**. Cumulative ratio of freedom from myocardial infarction among the four groups (*P* = 0.06). **b**. Cumulative ratio of freedom from all-death among the four groups (*P* < 0.01). **c**. Cumulative ratio of freedom from cardiac death among the four groups (*P* < 0.01). **d**. Cumulative ratio of freedom from repeated PCI among the four groups (*P* = 0.10)

The demographic data of the study population are presented in Table 2. Patients with DM and hypertension included more females and more CKD cases (both *P* < 0.01). Hypercholesterolemia was more likely in patients with hypertension alone, whereas b patients without DM and hypertension were most likely to be current smokers (both *P* < 0.01). Having a previous history of MI was highest in patients with DM alone (*P* < 0.01). Patients with DM and hypertension had the highest use of diuretics, beta blockers (BB) and angiotension receptor blockers (ARB) (all *P* < 0.01). Patients with hypertension alone used calcium channel blockers (CCB) and statins more frequently (both *P* < 0.01), but patients with DM alone had higher use of ace inhibitors (ACEI) (*P* < 0.01). Results of angiographic findings and clinical outcomes are shown in Table 3. Among angiographic findings, dual and triple vessel disease were found more frequently in patients with both DM and hypertension (*P* < 0.01), and these patients also had a larger number of treated vessels and lesions (both *P* < 0.01). No differences were found in invasive strategies among the four groups (*P* = 0.81). Among patient outcomes, patients with DM alone had the highest all-cause mortality and cardiovascular mortality rates (both *P* < 0.01); however, no differences were found in MI and repeated PCI rate between the four groups (*P* = 0.09 and *P* = 0.32, respectively). Figure 1 shows the cumulative rate of freedom from MI, cardiovascular death, all-cause death and repeated PCI among the four groups. Freedom from all-cause death and CV death were lowest in the DM alone group (both *P* < 0.01); however, no differences were found in MI and repeated PCI rate between the four groups (*P* = 0.06 and *P* = 0.10, respectively).

**Table 2:** Demography of study population and medications used after first time PCI

Variable	Study group				*P* value
	Control (*N* = 359)	DM alone (*N* = 178)	HT alone (*N* = 382)	DM and HT (*N* = 315)	
Gender					<0.01
F	61 (17.0%)	44 (24.7%)	110 (28.8%)	110 (34.9%)	
M	298 (83.0%)	134 (75.3%)	272 (71.2%)	205 (65.1%)	
CKD					<0.01
No	318 (88.6%)	132 (74.2%)	304 (79.6%)	196 (62.2%)	
Yes	41 (11.4%)	46 (25.8%)	78 (20.4%)	119 (37.8%)	
Hypercholesterolemia					<0.01
No	145 (40.4%)	98 (55.1%)	145 (38.0%)	156 (49.5%)	
Yes	214 (59.6%)	80 (44.9%)	237 (62.0%)	159 (50.5%)	
Current smoker					<0.01
No	192 (53.5%)	132 (74.2%)	241 (63%)	224 (71.1%)	
Yes	167 (46.5%)	46 (25.8%)	141 (37.0%)	91 (28.9%)	
Previous MI					<0.01
No	209 (58.2%)	98 (55.1%)	278 (72.7%)	222 (70.5%)	
Yes	150 (41.8%)	80 (44.9%)	104 (27.2%)	93 (29.5%)	
Stroke history					0.09
No	347 (96.7%)	164 (92.1%)	357 (93.5%)	293 (93.0%)	
Yes	12 (3.3%)	14 (7.9%)	25 (6.5%)	22 (7.0%)	
CABG history					0.67
No	358 (99.7%)	176 (98.9%)	380 (99.5%)	313 (99.4%)	
Yes	1 (0.3%)	2 (1.1%)	2 (0.5%)	2 (0.6%)	
Asprin					0.25
No	24 (6.7%)	21 (11.8%)	34 (8.9%)	26 (8.3%)	
Yes	335 (93.3%)	157 (88.2%)	348 (91.1%)	289 (91.8%)	
P2Y12 inhibitors					0.66
No	65 (18.1%)	35 (19.7%)	64 (16.8%)	49 (15.6%)	
Yes	294 (81.9%)	143 (80.3%)	318 (83.3%)	266 (84.4%)	
Diuretics					0.01
No	296 (82.5%)	131 (73.6%)	300 (78.5%)	229 (72.7%)	
Yes	63 (17.6%)	47 (26.4%)	82 (21.5%)	86 (27.3%)	
BB					0.10
No	213 (59.3%)	107 (60.1%)	213 (55.8%)	160 (50.8%)	
Yes	146 (40.7%)	71 (39.9%)	169 (44.2%)	155 (49.2%)	
CCB					<0.01
No	294 (81.9%)	126 (70.8%)	223 (58.4%)	197 (62.5%)	
Yes	65 (18.1%)	52 (29.2%)	159 (41.6%)	118 (37.5%)	
ACEI					<0.01
No	255 (71.0%)	127 (71.4%)	310 (81.2%)	254 (80.6%)	
Yes	104 (29.0%)	51 (28.7%)	72 (18.9%)	61 (19.4%)	
ARB					<0.01
No	316 (88.0%)	159 (89.3%)	267 (69.9%)	211 (67.0%)	
Yes	43 (12.0%)	19 (10.7%)	115 (30.1%)	104 (33.0%)	
Statin					<0.01
No	232 (64.6%)	134 (75.3%)	214 (56.0%)	210 (66.7%)	
Yes	127 (35.4%)	44 (24.7%)	168 (44.0%)	105 (33.3%)	
Fibrate					0.08
No	337 (93.9%)	170 (95.5%)	355 (92.9%)	283 (89.8%)	
Yes	22 (6.1%)	8 (4.5%)	27 (7.1%)	32 (10.2%)	

*DM alone* diabetes alone, *HT alone* hypertension alone, *DM and HT* both DM and hypertension, *Previous MI* history of previous myocardial infarction, *CABG history* history of coronary artery bypass graft, *CKD* chronic kidney disease, *P2Y12 inhibitor* P2Y12 receptor inhibitor of platelet, *BB* beta-blockers, *CCB* calcium channel blocker, *ACEI* angiotensin-converting enzyme inhibitor, *ARB* angiotensin receptor blocker

**Table 3:** Demography of angiographic findings and outcome

Variable	Study groups				*P* value
	Control (*N* = 359)	DM alone (*N* = 178)	HT alone (*N* = 382)	DM and HT (*N* = 315)	
Follow-up time (weeks)	173.8 ± 106.8	155.4 ± 104.8	168.8 ± 99.7	160.9 ± 99.0	0.17
Number of diseased vessels					<0.01^*^
Single vessel disease	206 (57.4%)	74 (41.6%)	186 (48.7%)	124 (39.4%)	
Dual vessel disease	93 (25.9%)	55 (30.9%)	107 (28.0%)	100 (31.8%)	
Triple vessel disease	60 (16.7%)	49 (27.5%)	89 (23.3%)	91 (28.8%)	
Mean of treated vessels	1.6 ± 0.8	1.9 ± 0.8	1.7 ± 0.8	1.9 ± 0.8	<0.01*
Mean of treated lesions	1.3 ± 0.7	1.4 ± 0.8	1.5 ± 0.8	1.6 ± 0.9	<0.01*
Type of intervention					0.81
Balloon angioplasty	122 (30.0%)	62 (15.2%)	125 (30.7%)	98 (24.1%)	
BMS deployment	144 (28.7%)	60 (11.8%)	171 (33.7%)	131 (25.8%)	
DES deployment	116 (25.8%)	68 (15.1%)	137 (30.4%)	129 (28.7%)	
Lesion location					0.62
LAD	249 (69.2%)	138 (78.0%)	288 (75.4%)	183 (58.1%)	
Lcx	163 (45.2%)	96 (54.2%)	183 (48.0%)	191 (60.6%)	
RCA	162 (45.0%)	99 (55.9%)	199 (52.0%)	175 (55.6%)	
SYNTAX score	10.8 ± 8.0	11.9 ± 7.9	10.1 ± 6.9	11.6 ± 8.5	<0.01*
LVEF	0.60 ± 0.13	0.55 ± 0.16	0.62 ± 0.13	0.59 ± 0.15	0.71
MI					0.09
yes	15 (4.2%)	14 (7.9%)	12 (3.1%)	17 (5.4%)	
no	344 (95.8%)	164 (92.1%)	370 (96.9%)	298 (94.6%)	
CV death					<0.01*
yes	20 (5.6%)	24 (13.5%)	13 (3.4%)	17 (5.4%)	
no	339 (94.4%)	154 (86.5%)	369 (96.6%)	298 (94.6%)	
All-cause death					<0.01*
yes	35 (9.8%)	36 (20.2%)	21 (5.5%)	22 (7.0%)	
No	324 (90.3%)	142 (79.8%)	361 (94.5%)	293 (93.0%)	
Re-PCI					0.32
yes	84 (23.4%)	51 (28.7%)	82 (21.5%)	73 (23.2%)	
No	275 (76.6%)	127 (71.4%)	300 (78.5%)	242 (76.8%)	

*BMS* bare metal stent, *DES* drug-eluting stent, *LAD* left anterior descending artery, *Lcx* left circumflex artery, *RCA* right coronary artery, *SYNTAX score* Synergy between Percutaneous Coronary Intervention with Taxus and Cardiac Surgery score, *LVEF* left ventricular ejection fraction, *MI* myocardial infarction, *Re-PCI* repeated percutaneous coronary intervention. ^*^: significant

Outcomes analysis and significant predictors of outcome evaluated by Cox proportion hazard model for MI, all-cause death, CV-death, and repeated PCI are shown in Table 4. Patients with DM alone carried the highest risk compared with the control group in terms of MI, CV death, all-cause death, and repeated PCI (Odds Ratio: 2.15, 2.25, 1.90, and 1.70, respectively, *P* < 0.01). Results of the Cox proportional hazard model revealed that previous MI and Syntax scores were predictors for MI (OR: 3.17 and 1.03, respectively), and use of statins reduced the risk of MI (OR: 0.43). Age, CKD, previous MI and stroke history were predictors for all-cause death (OR: 1.05, 1.89, 2.87, and 4.12, respectively), and use of BB and statins reduced the risk (OR: 0.47 and 0.35, respectively). Previous MI and stroke history, use of P2Y12 inhibitors, and syntax scores were all predictors of CV death (OR: 4.02, 1.89, 2.87, and 1.04, respectively), use of BB, ACEI, and statins reduced the risk of CV death (OR: 0.37, 0.33, and 0.32, respectively). Finally, smoking and use of BB were associated with repeated PCI procedures (OR: 1.48 and 1.56, respectively).

**Table 4:** Significant predictors of outcome in the Cox proportion hazard model for MI, All-cause death, CV-death, Repeated PCI

Variable	MI	All-death	CV-death	Repeated PCI
	HR^a^ (95% C.I.)	HR^a^ (95% C.I.)	HR^a^ (95% C.I.)	HR^a^ (95% C.I.)
Group				
Control	1.00 (Reference)	1.00 (Reference)	1.00 (Reference)	1.00 (Reference)
DM alone	2.15 (1.03–4.49)^*^	2.25 (1.19–4.26)^*^	1.90 (1.03–3.50)^*^	1.70 (1.19–2.44)^**^
HT alone	1.07 (0.49–2.33)	0.47 (0.19–1.14)	0.75 (0.37–1.53)	1.02 (0.75–1.38)
DM and HT	1.62 (0.79–3.33)	0.82 (0.39–1.72)	0.88 (0.45–1.71)	1.27 (0.92–1.75)
Age	–	1.05 (1.02–1.07)^**^	–	–
CKD	–	1.89 (1.06–3.36)^*^	–	–
Smoking	–	–	–	1.48 (1.16–1.89)^**^
Previous MI	3.17 (1.80–5.57)^**^	2.87 (1.65–4.99)^**^	4.02 (2.38–6.8)^**^	–
Stroke	2.05 (0.81–5.22)	4.12 (2.04–8.32)^**^	1.89 (0.95–3.76)^*^	–
Diuretics	–	–	1.27 (0.74–2.17)	–
Aspirin		–	1.17 (0.55–2.47)	–
P2Y12 inh		–	2.87 (1.04–7.95)^*^	–
BB	–	0.47 (0.26–0.85)^*^	0.37 (0.22–0.64)^**^	1.56 (1.24–1.98)^**^
CCB	–	–	–	–
ACEI	–	–	0.33 (0.18–0.67)^**^	–
ARB	–	–	–	–
Statin	0.43 (0.23–0.81)^**^	0.35 (0.17–0.70)^**^	0.32 (0.17–0.62)^**^	–
Syntax score	1.03 (1.00–1.06)^*^	1.02 (0.99–1.04)	1.04 (1.01–1.06)^**^	1.00 (0.98–1.01)

*DM alone* diabetes alone, *HT alone* hypertension alone, *DM and HT* both DM and hypertension

*CKD* estimated glomerular filtration rate <60 ml/min, *Previous MI* history of previous myocardial infarction, *P2Y12 inh* P2Y12 receptor inhibitor of platelet, *Beta B* beta-blockers, *CCB* calcium channel blocker, *ACEI* angiotensin-converting enzyme inhibitor, *ARB* angiotensin receptor blocker, *Syntax score* Synergy between Percutaneous Coronary Intervention with Taxus and Cardiac Surgery score

^*^ *P* < 0.05, ^**^ *P* < 0.01. ^a^HR was adjusted for confounding

RMI Model: y = βdummyDH1 + βdummyDH2 + βdummyDH3 + βMI + βstroke + βstatin + βsyntax

All-death model: y = βdummyDH1 + βdummyDH2 + βdummyDH3 + βage + βCKD + βMI + βstroke + βbetab + βstatin + βsyntax

CV-death model: y = βdummyDH1 + βdummyDH2 + βdummyDH3 + βMI + βstroke + βdiuretics + βbetab + βACEI + βstatin + βsyntax

Repeated-PCI model: y = βdummyDH1 + βdummyDH2 + βdummyDH3 + βMI + βsmoking + βbetab + βsyntax

## Discussion

In the present study, patients with coronary artery disease receiving percutaneous coronary intervention had the highest rate of all-cause mortality and CV mortality compared to patients without DM and hypertension, patients with both DM and hypertension, and those with hypertension alone and DM alone. However, no difference were found in de novo MI and repeated PCI between the four groups. Age, CKD, previous MI and stroke history were predictors for all-cause death. Previous MI and stroke history, use of P2Y12 inhibitors, and syntax scores were all predictors for CV death. Previous MI history and syntax scores were predictors for MI, and smoking and use of BB were associated with repeated PCI procedures. While statin use reduced the risk of MI, CV death and all-cause death, BB reduced the risk of CV death and all-cause death, and CEI reduced CV death.

Results of the present study also showed that patients with DM alone as well as patients with both DM and hypertension had a higher prevalence of elevated serum creatinine levels and CKD, and for this reason, the use of diuretics was also higher than in the other groups. On the other hand, for renal function, patients with DM and hypertension had more elevated serum creatinine levels and increased prevalence of CKD than patients with DM alone (*P* < 0.04 and *P* < 0.007, respectively).

Given that hypertension seems to have an adverse effect on renal function in DM patients, hypotensive agents with more potency such as ARB were used more frequently than ACEI for BP control in patients with DM and hypertension (33% vs. 19.4%, *P* < 0.001). In contrast, compared with patients with DM alone, or patients with both DM and hypertension, ACEI were used more often than in the other two groups because of the higher prevalence of previous MI. In the present study, patients with DM alone had the lowest rate of hypercholesterolemia, and statin use was the lowest in this group compared to the other groups. Although statin use when LDL is less than 70 mg/dL has been found to improve cardiovascular outcomes in CAD patients after ACS [16], whether statin under-usage led to the poor outcomes in DM patients in this study remains to be clarified. In comparison with patients with DM only, patients with hypertension alone and those with both DM and hypertension used statins, high potency hypotensive agents such as calcium channel blockers (CCB) and ARB more frequently, which may have led to a better prognosis.

No differences were found between groups regarding lesion location and type of intervention such as balloon angioplasty, bare metal stent deployment, or drug eluting stent deployment. However, patients with both DM and hypertension and those with DM alone had a greater prevalence of dual vessel disease and triple vessel disease; the SYNTAX scores were also higher than for patients without DM and hypertension, and hypertension alone. Besides, based on optical coherence tomography (OCT) study, the factors implicated with culprit plaque rupture were different depending on clinical presentations. Hypertension was the only predictor for ST-segment elevation myocardial infarction (STEMI), while advancing age, DM, and hyperlipidemia were the predictors for non-ST segment elevation myocardial infarction (NSTEMI) and unstable angina (US) [17]. On the other hand, in terms of the prevalence of multi-vessel disease, number of diseased vessels and number of treated lesions, no differences were found between patients with DM alone and patients with hypertension alone (*P* = NS). Compared with patients with hypertension alone, patients with both DM and hypertension had a significantly higher risk of developing multi-vessel disease (*P* = 0.04); however, compared to patients with DM alone, patients with both DM and hypertension did not have a significant risk of developing multi-vessel disease (*P* = 0.65). Comorbidity with DM in hypertension patients might have the additional risk of multi-vessel disease in comparison with patients with hypertension alone. In terms of treated lesions, no differences were found between patients with hypertension alone, and patients with both DM and hypertension (1.5 ± 0.8 vs 1.6 ± 0.9, *P* = NS). However, patients with DM alone had fewer treated lesions than patients with both DM and hypertension (1.4 ± 0.8 vs 1.6 ± 0.9, *P* < 0.05). Although the prevalence rate of multi-vessel disease of DM patients was not different from patients with both DM and hypertension, the DM alone patients received fewer procedures providing aggressive revascularization.

Evidence-based medicine has shown that hypotensive agents and statin provide target-organ protection [18–23]. In the present study, patients with DM alone had the highest rates of MI, all-cause mortality and CV mortality compared to the other groups. Similarly, when compared with patients with DM and hypertension, patients with DM alone had a significantly increased risk of MI, CV death and all-death (*P* < 0.001, *P* < 0.002, and *P* < 0.006, respectively). However, no significant differences were noted in terms of MI, CV death and all-death between patients with hypertension alone and patients with both DM and hypertension (*P* = 0.50, *P* = 0.60, and *P* = 0.41, respectively). This may be due to the fact that patients DM alone had a higher rate of previous MI, less use of statins, and less use of more potent hypotensive agents. Moreover, a less aggressive invasive strategy may also have played an important role. Hypertensive patients with or without coexisting DM have a better prognosis because of greater use of statins and potent hypotensive agents, and a more aggressive invasive strategy.

### Study limitations

First, intensity of medical control such as tight blood glucose control rate and BP control rate were not surveyed in this study, adherence to drug therapy was not evaluated in this study. Second, functional evaluations of the atherosclerotic lesions, such as plaque compositions analysis and fraction flow reserve (FFR) measurement, were not used, which may also have had an impact on the index PCI. Third, the case number of DM alone patients was smaller than for the other groups, which may have affected the power of this study. Fourth, this study is small and thus underpowered to determine the effect of hypertension on CVD outcomes after PCI, given smaller effect size and the need for a longer duration of follow-up. Finally, since this is a prospective cohort study, whether both aggressive medical treatment and invasive strategy could improve outcome in DM alone patients a remains to be clarified by large randomized clinical trials.

## Conclusions

Patients with DM alone have higher mortality than patients without DM and hypertension, with both DM and hypertension, and with hypertension alone. Comorbid hypertension appears not to increase risk in DM patients, whereas comorbid DM appears to increase risk in hypertensive patients.

## Abbreviations

ACEI: Angiotensin-converting enzyme inhibitor
ARB: Angiotensin receptor blocker
BB: Beta-blockers
BMI: Body mass index
BMS: Bare-metal stent
CABG: Coronary artery bypass graft
CAD: Coronary artery disease
CAP: Central aortic pressure
CCB: Calcium channel blockers
CDP: Central aortic diastolic pressure
CKD: Chronic kidney disease
CSP: Central aortic systolic pressure
CV mortality: Cardiovascular mortality
DES: Drug-eluting stent
eGFR: Estimated glomerular filtration rate
FFR: Fraction flow reserve
HbA1c: Hemoglobin A1C
HDL-C: High-density lipoprotein-cholesterol
LAD: Left anterior descending artery
Lcx: Left circumflex artery
LDL-C: Low-density lipoprotein- cholesterol
LVEF: Left ventricular ejection fraction
MI: Myocardial infarction
OCT: Optical coherence tomography
OPD: Outpatient department
PCI: Percutaneous coronary intervention
RCA: Right coronary artery
SYNTAX score: Synergy between percutaneous coronary intervention with taxus and cardiac surgery score
TG: Triglyceride

## References

[1] Williams DO, Abbott JD, Kip KE (2006). DEScover investigators. Outcomes of 6906 patients undergoing percutaneous coronary intervention in the era of drug-eluting stents: report of the DEScover Registry. Circulation.

[2] Lazzeri C, Valente S, Chiostri M, Attana P, Picariello C, Gensini GF (2012). Impact of hypertension on short and long- term prognoses in patients with ST elevation myocardial infarction and without previously known diabetes. Heart Vessels.

[3] Cecchi E, D’Alfonso MG, Chiostri M, Parigi E, Landi D, Valente S (2014). Impact of hypertension history on short and long-term prognosis in patients with acutemyocardial infarctioon treated with percutaneous angioplasty: comparison between STEMI and NSTEMI. High Blood Press Cardiovasc Prev.

[4] Lopez Minguez JR, Fuentes ME, Doblado MI, Merchán A, Martińez A, González R (2003). Prognostic role of systemic hypertension and diabetes mellitus in patients with unstable angina undergoing coronary stenting. Rev Esp Cardiol.

[5] Hoebers LP, Claessen BE, Woudstra P, DVries JH, Wykrzykowska JJ, Vis MM (2014). Long-term mortality after primary percutaneous coronary intervention for ST-segment elevation myocardial infarction in patients with insulin-treated versus non-insulin–treated diabetes mellitus. Eurointervention.

[6] Klempfner R, Elis A, Matezky S, Keren G, Roth A, Finkelstein A (2015). Temporal trends in management and outcome of diabetic and nn-diabetic patients with acute coronary syndrome (ACS): residual risk of long-term mortality persists: insight from the ACS Israeli Survey (ACSIS) 2000–2010. Int J Cardiol.

[7] Park KH, Ahn Y, Jeong MH, Chae SC, Hur SH, Kim YJ (2012). Different impact of diabetes mellitus on in-hospital and 1-year mortality in patients with acute myocardial infarction intervention: results from the Koran Acute Myocardial Infarction Registry. Korean J Intern Med.

[8] Jensen LO, Maeng M, Thayssen P, Tilsted HH, Terkelsen CJ, Kaltoft A (2012). Influence of diabetes on clinical outcomes following primary percutaneous coronary intervention in patients with ST-segment elevation myocardial infarction. Am J Cardiol.

[9] Kahn MB, Cubbon RM, Mercer B, Wheatcroft AC, Gherardi G, Aziz A (2012). Association of diabetes with increased all-cause mortality following primary percutaneous coronary intervention for ST-segmenty elevatin myocardial infarction in the contemporary era. Diab Vas Dis Res.

[10] Lee MG, Jeong MH, Lee KH, Park SH, Sim DS, Yoon HJ (2012). Prognostic impact of diabetes and hypertension for mid-term outcome of patients with acute myocardial infarction who underwent percutaneous coronary intervention. J Cardiol.

[11] Mathew V, Gersh BJ, Williams BA, Laskey WK, Willerson JT, Tilbury RT (2004). Outcomes in patients with diabetes mellitus undergoing percutaneous coronary intervention in the current era: a report from the Prevention of ReStenosis with tranilast and its outcome (PRESTO) trial. Circulation.

[12] Laskey WK, Selzer F, Vlachos HA, Johnston J, Jacobs A, King SB (2002). Comparison of in-hospital and one-year outcomes in patients with and without diabetes mellitus undergoing percutaneous catheter intervention (from the National Heart, Lung and Blood Institute Dynamic Registry). Am J Cardiol.

[13] Serruys PW, Morice MC, Kappetein AP, Colombo A, Holmes DR, Mack MJ (2009). SYNTAX investigators. Percutaneous coronary intervention versus coronary-artery bypass grafting for severe coronary artery disease. N Engl J Med.

[14] American Diabetic Association. Diagnosis and classification of Diabetes Mellitus. Diabetes Care. 2013;36 Supplement 1:S67-S74. 10.2337/dc13-S067 PMC3537273 23264425

[15] National Kidney Foundation, Kidney Disease Outcome Quality Initiative (K/DOQI) (2003). Clinical practice guidelines for bone metabolism and disease in chronic kidney disease. Am J Kid Dis.

[16] Cannon CP, Blazing MA, Giugliano RP, McCagg A, White JA, Theroux P (2015). Ezetimibe added to statin therapy after acute coronary syndromes. N Engl J Med.

[17] Iannaccone M, Quadri G, Taha S, D’Ascenzo F, Montefusco A, Omede’ P (2016). Prevalence and predictors of culprit plaque rupture at OCT in patients with coronary artery disease: a meta-analysis. Eur Heart J Cardiovasc Imaging.

[18] Poulter NR, Wedel H, Dahlöf B, Server PS, Beevers DG, Caulfield M (2005). Role of blood pressure and other variables in the differential cardiovascular event rates noted in the Anglo-Scandinavian Cardiac Outcomes Trial-Blood Pressure Lowering Arm (ASCOT-BPLA). Lancet.

[19] Sever PS, Dahlöf B, Poulter NR, Wedel H, Beevers G, Caulfield M (2003). Prevention of coronary and stroke events with atorvastatin in hypertensive patients who have average or lower-than-average cholesterol concentrations, in the Anglo-Scandinavian Cardiac Outcomes Trial--Lipid Lowering Arm (ASCOT-LLA): a multicentre randomized controlled trial. Lancet.

[20] Deckers JW, Goedhart DM, Boersma E, Briggs A, Bertrand M, Ferrari R (2006). Treatment benefit by perindopril in patients with stable coronary artery disease at different levels of risk. Eur Heart J.

[21] Yusuf S, Sleight P, Pogue J, Bosch J, Davies R, Dagenais G (2000). Effects of an angiotensin-converting-enzyme inhibitor, ramipril, on cardiovascular events in high-risk patients. The Heart Outcomes Prevention Evaluation Study Investigators. N Engl J Med.

[22] Heart Protection Study collaborativer Group (2002). MRC/BHF Heart Protection Study of cholesterol lowering with simvastatin in 20,536 high-risk individuals: a randomised placebo-controlled trial. Lancet.

[23] The Long-Term Intervention with Pravastatin in Ischaemic Disease(LIPID) Study Group.Prevention of cardiovascular events and death with pravastatin in patients with coronary heart disease and a broad range of initial cholesterol levels. N Engl J Med. 1998;339(19):1349-57. 10.1056/NEJM199811053391902 9841303

